# 

**Published:** 1892-06-11

**Authors:** 


					.Trice twopence.
I MB, Vol. Xll.-June 11th, 1892.
at the General Post Office as a Newspawr for
^mission in the United Kingdom and Abroad, l
Per Annum, 8s. 6d., post free.
Monthly Farts, 6d.; post free, 8d.
Ditto per Annum, 8s., post free.
No. 298. Vol. XTT.] Saturday,
June 11th, 1892.
[Twopence.
SPECIAL HOSPITAL SUNDAY NUMBER.

				

